# Use of Double-Parallel Oxygenators for Hypercapnia During Veno-Venous Extracorporeal Membrane Oxygenation: A Case Report

**DOI:** 10.7759/cureus.79757

**Published:** 2025-02-27

**Authors:** Ryo Miyagawa, Jun Hamaguchi, Keiji Aibara, Masayuki Kamochi, Keiki Shimizu

**Affiliations:** 1 Department of Emergency and Intensive Care Medicine, University of Occupational and Environmental Health, Kitakyushu, JPN; 2 Department of Emergency and Critical Care Medicine, Extracorporeal Membrane Oxygenation (ECMO) Center, Tokyo Metropolitan Tama Medical Center, Tokyo, JPN

**Keywords:** covid-19, double-parallel oxygenators, ecmo dependence, hypercapnia, v-v ecmo

## Abstract

A 76-year-old man arrived at our hospital post-intubation for coronavirus disease, and veno-venous extracorporeal membrane oxygenation (V-V ECMO) was performed for hypoxemia the same day. Although renal replacement therapy was introduced for anuria due to bacterial pneumonia, the patient became fluid-overloaded. In addition to the effects of fluid overload and pneumonia, the ventilator was adjusted to a lung rest strategy, making oxygen delivery and carbon dioxide (CO_2_) removal almost entirely dependent on ECMO. Even with high-flow sweep gas, CO_2_ removal was difficult because of the relatively large body surface area, hypercapnia renal compensation difficulties due to acute kidney injury, increased CO_2_ production because of infection, and increased membrane lung shunting secondary to blood coagulation disorders. Therefore, we used double-parallel oxygenators for the hypercapnia and reduced the sweep-gas flow because of the increased membrane lung area. These results suggest that double-parallel oxygenator use provides effective management for refractory hypercapnia during ECMO.

## Introduction

When adequate gas transfer cannot be maintained on a mechanical ventilator for severe respiratory failure, veno-venous extracorporeal membrane oxygenation (V-V ECMO) is introduced as an adjunctive therapy. V-V ECMO would reduce lung injury during respiratory failure and improve prognosis using a mechanical ventilator in the lung-rest setting [1,2]. Using ECMO, carbon dioxide (CO_2_) elimination is mainly determined by the membrane lung surface area and sweep gas flow rate. When respiratory acidosis does not improve with an increased sweep gas flow rate, a double lung technique is utilized to increase the membrane lung area by using double-parallel oxygenators [3]. We describe a case of severe respiratory failure complicated by COVID-19 pneumonia and bacterial pneumonia, which was treated with V-V ECMO; however, CO_2_ removal was difficult, and its clearance was successfully achieved using double-parallel oxygenators.

## Case presentation

A 76-year-old man with a history of lower extremity arteriosclerosis obliterans was admitted to our hospital and had been previously treated for respiratory failure associated with coronavirus disease 2019 (COVID-19). Due to a worsening respiratory condition after three days, he was referred to our hospital after intubation for COVID-19, and V-V ECMO was initiated for hypoxemia the same day.

**Figure 1:**
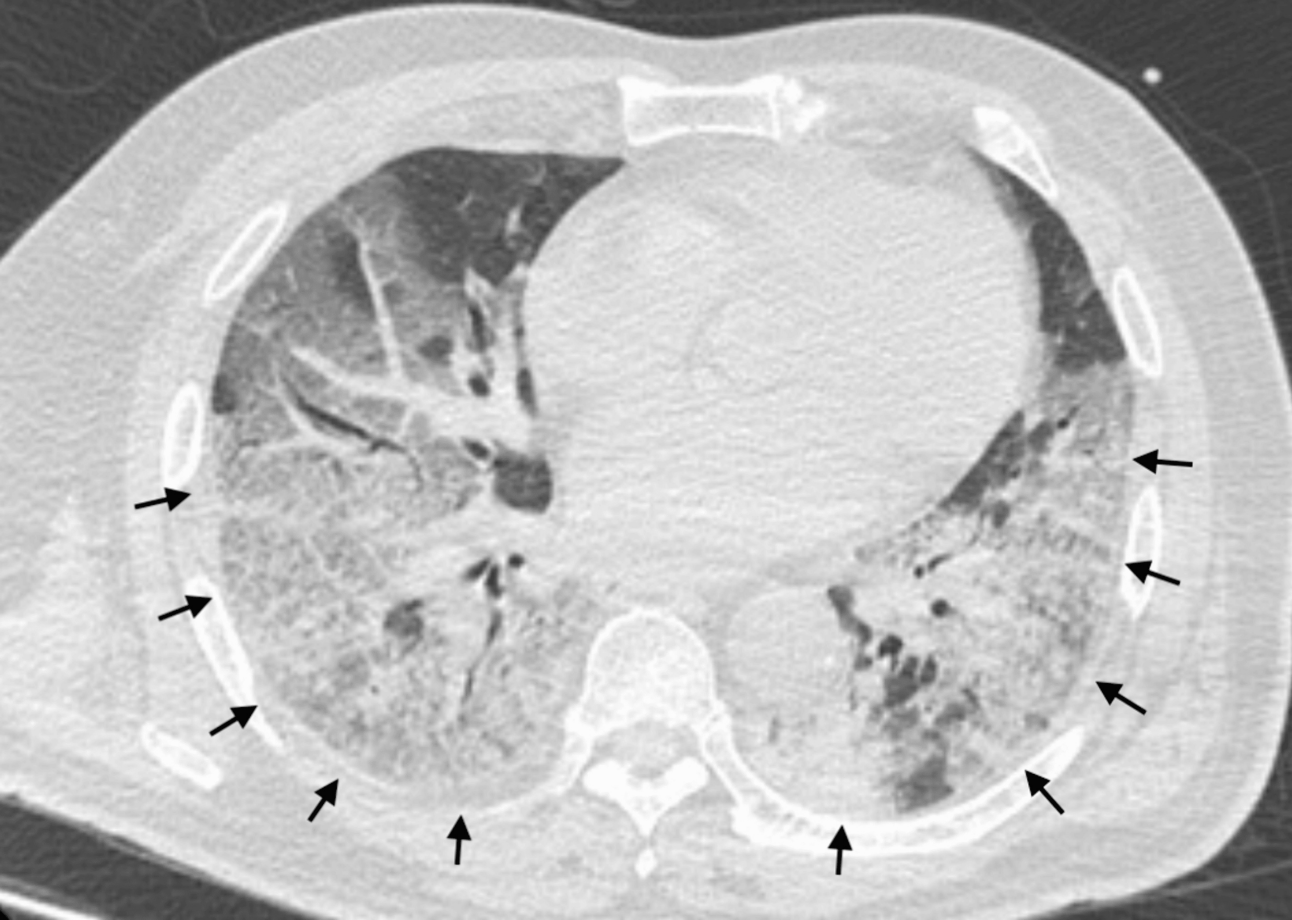
Chest computed tomography from the referral hospital Computed tomography revealed extensive ground-glass opacities, predominantly in the periphery of both lungs.

The patient had a relatively large body surface area (BSA) (height: 170 cm, body weight: 86.0 kg, body mass index: 29.8 kg/m^2^, and BSA: 1.98 m^2^). Upon admission, the patient had the following vital signs: a Glasgow Coma scale score of E1VTM1 (on sedation, analgesia and neuromuscular block), a respiratory rate of 20 breaths/min, a heart rate of 101 beats/min, a blood pressure of 152/63 mmHg, a body temperature of 37.2℃, and an oxygen saturation of 92% (on mechanical ventilation). The ventilator settings were as follows: a fraction of inspired oxygen (FiO_2_) of 1.0, positive end-expiratory pressure (PEEP) of 14 cmH_2_O, and peak inspiratory pressure (PIP) of 28 cmH_2_O (tidal volume of 420 mL and lung compliance of 30 mL/cmH_2_O). On admission to our hospital, arterial blood gas analysis (under the ventilator settings described above) revealed a mixed acidosis with a pH of 7.246, a partial pressure of carbon dioxide (PaCO_2_) of 53.9 mmHg, a partial pressure of oxygen (PaO_2_) of 72.3 mmHg, and a bicarbonate level of 22.6 mmol/L. The laboratory test findings showed an increased inflammatory response (white cell count: 13300 /µL; reference range: 3,300-8,600 /µL and C-reactive protein level: 17.2 mg/dL; reference range: 0.0-0.14 mg/dL), a prolonged activated partial thromboplastin time of 64.6 s (reference range: 23.5-31.5 s), and a low D-dimer level (0.8 μg/dL; reference range: 0.0-1.0 μg/dL) because of heparin. On the day of admission, chest radiography and computed tomography performed at the hospital revealed extensive ground-glass opacities, predominantly in the periphery of both lungs (Figure 1).

**Figure 2:**
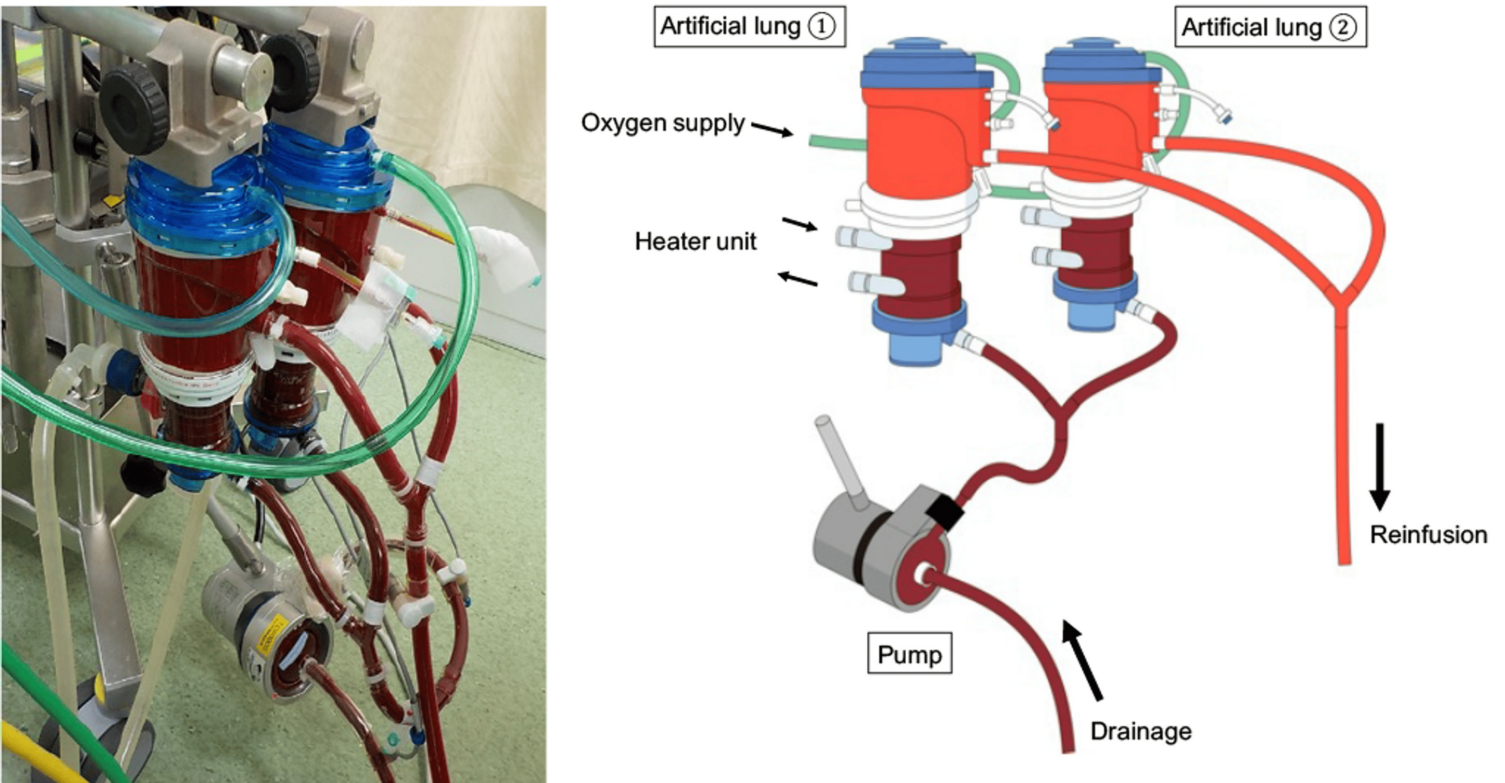
Double-parallel oxygenator (X+30 d) pump* *Rotaflow®︎（GETINGE. Germany) Lung: Mera Excelung®︎ (Senko Medical Institute, Japan)

Our staff conducted a site visit, and the patient was transported on a mechanical ventilator using the mobile intensive care unit. The respiratory status of the patient was re-evaluated after admission to our hospital. He was given muscle relaxants and lung protective ventilation but was not placed in the prone position. The reason why he was not placed in the prone position was that hemodynamics had become progressively unstable from the time of admission to our hospital. The same day, we introduced V-V ECMO because the patient displayed a PaO_2_/FiO_2_ ratio of 72.3 (FiO_2_: 1.0), a Murray score of 3.5 points, and a RESP (respiratory extracorporeal membrane oxygenation survival prediction) score of 1. The RESP score is a scoring system that predicts survival and discharge rates from data at the time of ECMO introduction for respiratory failure in general [4]. A 25Fr cannula was inserted by echo-guided puncture into the right internal jugular vein for drainage, while a 21Fr cannula was placed by echo-guided puncture in the right femoral vein as a return. The positions of the cannulae were finalized with X-rays. V-V ECMO (Cardiohelp system, Maquet Getinge Cardiopulmonary AG, Rastatt, Germany) was established at a blood flow rate of 4.12 L/min and rotation speed of 3000 rpm, and the mechanical ventilator was set to a lung rest setting (FiO_2_: 0.4, PEEP: 15 cmH_2_0, PIP: 20 cmH_2_0, and f: 5/min). Continuous intravenous unfractionated heparin and tocilizumab were administered to treat COVID-19. Hemodynamics began deteriorating a few hours after the ECMO initiation, and vasopressors were used due to the effects of the latter. Immediately after admission, bronchoscopy revealed a large amount of purulent sputum every day. Streptococcus pyogenes was detected in the admission sputum culture, and S. pyogenes was detected in blood cultures on day 3 of admission, which was not found on admission. The hyperpermeability cause was determined to be a fulminant hemolytic streptococcal infection associated with pneumonia. Circulation gradually improved after antimicrobial therapy was initiated on day 3 of admission, although the patient’s body weight increased by +15 kg. Anuria and progressive metabolic acidosis associated with acute kidney injury were present. Therefore, continuous renal replacement therapy (CRRT) was initiated on day 3 of admission, and the body weight was reduced to +1 kg at the time of admission on day 13. However, on day 14 of admission, he developed ventilator-associated pneumonia caused by extended-spectrum β-lactamase-producing Proteus mirabilis. Additionally, Citrobacter koseri was detected in blood cultures on days 20 and 29, and Enterococcus faecalis was detected in blood cultures on days 25 and 27. Due to the repeated infections, the patient developed a fluid overload of >10 L again. Even with high-flow sweep gas, CO_2_ removal is difficult because of the relatively large BSA, difficulty in renal hypercapnia compensation due to acute kidney injury, increased CO_2_ production caused by infections, and increased membrane lung shunts due to membrane lung thrombosis secondary to blood coagulation disorders. The sweep gas flow rate was 4 L/min immediately after V-V ECMO introduction but gradually increased to 9 L/min on day 24 of admission. On day 27 of admission, PaCO_2_ was 46.5 mmHg with a sweep gas flow of 9 L/min. The consideration of weaning from ECMO and the change in ventilator settings led to an improvement in PaCO_2_ to 40.2 mmHg on day 31 of admission. Nevertheless, high-flow sweep gas was still required with the ventilator setting (Table 1). Even when the sweep gas flow rate is increased, the efficiency of CO_2_ clearance decreases, and high-flow sweep gas poses a risk of air embolism. ECMO blood flow rate has little effect on CO_2_ clearance, but increasing the membrane surface area improves CO_2_ clearance [5,6]. Therefore, even with an increased sweep gas flow, no further CO_2_ clearance was expected. Circuit exchange was necessary due to membrane lung deterioration on days 17 and 23 of admission, and massive blood transfusions were performed before the circuit exchanges. We decided to change double-parallel oxygenators as they increased the membrane area to enhance CO2 clearance and prolong circuit life on day 31 of admission (Figure 2).

**Table 1:** Effects of the double-lung system ECMO: extracorporeal membrane oxygenation, FiO_2_: a fraction of inspired oxygen, PIP: peak inspiratory pressure, PEEP: positive end-expiratory pressure, TV: tidal volume, PaCO_2_: partial pressure of carbon dioxide, PaO_2_: partial pressure of oxygen, EIT: electrical impedance tomography,  Ptp: transpulmonary pressure

Date of admission	X	X+22	X+26	X+30 (one-lung)	X+30 (double-lung)	X+34
							ECMO settings						
Flow (L/min)	4.12	4.02	4.31	4.64	4.74	4.75
Sweep gas (L/min)	4	7	9	9	5(2.5+2.5)	4(2+2)
Ventilator settings						
FiO_2_	0.4	0.4	0.4	0.4	0.4	0.4
PIP (cmH_2_O)	20	25	25	32*	32*	32
PEEP (cmH_2_O)	15	15	15	14*	14*	14
TV (mL)	112	80	80	250	250	250
Arterial blood gas						
pH	7.215	7.259	7.284	7.36	7.388	7.365
PaCO_2_ (mmHg)	64.1	53.2	46.5	40.2	35	37.4
PaO_2_ (mmHg)	65	65.3	48.7	77.9	96	132
*We used EIT and transpulmonary pressure to change ventilator settings for ECMO weaning on day X+29 (ΔPtp=8.9 cmH_2_O, Anterior/Posterior ratio; 2.70→2.03, Static lung compliance 12→17 mL/cmH_2_O, TV 190→250 mL).						

At the same time, we altered the ECMO configurations to control for persistent bacteremia. A 25Fr cannula was placed by puncture in the left femoral vein for drainage, and a 21Fr cannula was inserted by puncture in the left internal jugular vein as a return. The sweep gas flow rate was reduced to 5 L/min (2.5+2.5) immediately after changing to double-parallel oxygenators because the efficiency of CO2 clearance improved (Table 1).

After the configuration change, the patient was weaned off ECMO on day 36 of admission because the infections were controlled and he was actively dehydrated. The patient was weaned off the ventilator on day 122 after admission.

## Discussion

V-V ECMO is considered for severe respiratory failure, which is difficult to manage with mechanical ventilation, as in this case. Since the 2009 influenza (H1N1) pandemic, evidence has demonstrated that V-V ECMO would improve outcomes in patients with severe respiratory failure [7-9]. Even during the COVID-19 pandemic, V-V ECMO was introduced for many patients with severe respiratory failure. Data from Japan demonstrate that as patients age increases, their prognosis worsens [10]. Therefore, age is an important indication criterion. Although the patient was in his 70s, the decision was made to initiate V-V ECMO because he independently conducted activities of daily living, and his severe respiratory failure secondary to COVID-19 was not accompanied by multiple organ failure. V-V ECMO assists with oxygenation and ventilation. Ventilation is determined by the membrane lung area and sweep gas flow rate. Hypercapnia can cause increased intracranial pressure, pulmonary hypertension, decreased cardiac contractility, decreased renal blood flow, and endogenous catecholamine release [11]. Therefore, if CO_2_ clearance is inadequate during ECMO management despite a sufficiently high flow sweep gas, it is necessary to change to a larger membrane lung or use double-parallel oxygenators to increase the membrane area. Melro reported CO_2_ clearance in swine models using double-parallel oxygenators and found that their use significantly lowered PaCO_2_ compared with the use of a single oxygenator [12]. However, PaCO_2_ can be controlled by adjusting the sweep-gas flow rate in most cases, and it is rare to report a case in which double-parallel oxygenators were used [3]. In this case, most of the ventilation was dependent on ECMO because the baby lung was affected by fluid overload and pneumonia, and the mechanical ventilator was on the lung rest setting. Additionally, even with high-flow sweep gas, CO_2_ removal is difficult because of the relatively large BSA in Asians (1.975 m^2^), difficulty with hypercapnia renal compensation as a result of acute kidney injury during CRRT, increased CO_2_ production due to infection, and increased membrane lung shunts secondary to thrombosis in the membrane lung due to blood coagulation disorders. Therefore, double-parallel oxygenators were used (Figure 2). Additionally, we expected that increasing the oxygenator number to two would extend the circuit life and avoid increased transfusion volume and complications due to circuit replacement. Therefore, the total sweep-gas flow rate was immediately reduced from 9 L/min to 2.5 L/min for each oxygenator, although the circuit life was not extended (Table 1). We changed the ECMO configuration to control persistent bacteremia in conjunction with changing the double-parallel oxygenators. The configuration change was successful in infection control and allowed aggressive dehydration; further, the sweep gas flow gradually decreased as the tidal volume steadily increased.

## Conclusions

Carbon dioxide removal is rarely difficult in typical V-V ECMO management. In this case, most of the gas exchange was dependent on ECMO. In addition, even with high-flow sweep gas, CO_2_ removal is difficult because of the relatively high BSA, difficulty with renal hypercapnia compensation due to acute kidney injury, increased CO_2_ production due to infection, and increased membrane lung shunts secondary to thrombosis in the membrane lung due to coagulopathy. Therefore, we treated a patient who required high flow sweep gas with double-parallel oxygenators during V-V ECMO. It is rare to report a case in which double-parallel oxygenators were used. The use of double-parallel oxygenators during ECMO for difficult-to-control hypercapnia can provide effective management.
